# A Cell-Based High-Throughput Screen Addressing 3′UTR-Dependent Regulation of the *MYCN* Gene

**DOI:** 10.1007/s12033-014-9739-z

**Published:** 2014-02-11

**Authors:** Viktoryia Sidarovich, Valentina Adami, Alessandro Quattrone

**Affiliations:** 1 Laboratory of Translational Genomics, Centre for Integrative Biology, University of Trento, via delle Regole 101, 38123 Trento, Italy; 2 High-Throughput Screening Core Facility, Centre for Integrative Biology, University of Trento, via delle Regole 101, 38123 Trento, Italy

**Keywords:** Post-transcriptional control, Screening, Luciferase, 3′UTR, MYCN, Neuroblastoma

## Abstract

Neuroblastoma is the most common extracranial solid tumor of infancy. Amplification of *MYCN* oncogene is found in approximately 20 % of all neuroblastoma patients and correlates with advanced disease stages, rapid tumor progression, and poor prognosis, making this gene an obvious therapeutic target. However, being a transcriptional factor MYCN is difficult for pharmacological targeting, and there are currently no clinical trials aiming MYCN protein directly. Here we describe an alternative approach to address deregulated MYCN expression. In particular, we focus on the role of a 3′ untranslated region (3′UTR) of the *MYCN* gene in the modulation of its mRNA fate and identification of compounds able to affect it. The luciferase reporter construct with the full length *MYCN* 3′UTR was generated and subsequently integrated in the CHP134 neuroblastoma cell line. After validation, the assay was used to screen a 2000 compound library. Molecules affecting luciferase activity were checked for reproducibility and counter-screened for promoter effects and cytotoxic activity resulting in selection of four hits. We propose this cell-based reporter gene assay as a valuable tool to screen chemical libraries for compounds modulating post-transcriptional control mechanisms. Identification of such compounds could potentially result in development of clinically relevant therapeutics for various diseases including neuroblastoma.

**Electronic supplementary material:**

The online version of this article (doi:10.1007/s12033-014-9739-z) contains supplementary material, which is available to authorized users.

## Introduction

Post-transcriptional regulation is a rapid and dynamic cellular process allowing for fast changes in protein levels in a transcription-independent way. Post-transcriptional control processes comprise various mechanisms such as mRNA processing (polyadenylation, capping, splicing), mRNA export and localization, mRNA turnover, and translation efficiency. These processes are known to be mediated by RNA-binding proteins (RBPs) and non-coding RNAs that bind to specific sequences or secondary structures, typically within mRNA 5′UTRs and 3′UTRs [1]. In general, the degree of “uncoupling” between transcriptome (total mRNA quantity) and translatome (quantity of mRNA loaded on polysomes) abundance is very high [2], indicating that the variations induced by post-transcriptional control mechanisms are significant for final shaping of the phenotype.

If a therapeutically relevant protein is derived from an mRNA with a predominant post-transcriptional regulation, specific targeting of these mechanisms can yield important implications [3]. For example, the human oncogene MDM2 has been recently shown to bind to AU-rich elements (AREs) in the mRNA 3′UTR of yet another oncogene, MYCN, stabilizing it [4]. Accordingly, two small-molecule antagonists of MDM2, Nutlin-3, and MI-63, might be of particular interest in treating MYCN-dependent diseases, such as high-risk neuroblastoma [5]. Indeed, Nutlin is currently enrolled as potential therapeutics in preclinical phase of testing.

While understanding of post-transcriptional regulation mechanisms has increased, this knowledge has not been accompanied by similar progresses in identifying compounds capable of modulating mRNA fate through interference with these mechanisms. Reporter cell-based assays offer a versatile and technically simple way to test molecules in high-throughput format [6]. However, until recently this kind of assay has been mainly focused on the identification of promoter-targeting compounds [7]. A similar approach in which a reporter gene is flanked by the 5′UTR and/or 3′UTR of a gene of interest could become a valuable tool for identification of molecules targeting post-transcriptional control mechanisms [8–10].

Neuroblastoma (NB) is a tumor derived from primitive cells of the sympathetic nervous system and is the most common extracranial solid tumor in childhood. The hallmark of NB is heterogeneity of clinical behavior, which ranges from spontaneous regression to rapid progression and death despite multimodal intensive therapy [11]. Being a relatively rare disease, NB disproportionally accounts for approximately 15 % of children cancer related deaths [12]. Therefore, new therapies are urgently needed.

Clinical and biological features, such as age at diagnosis, disease stage, numerical (ploidy) and structural chromosomal alterations (*MYCN* gene amplification; 1p, 3p, 11q deletions; 17q gain), are used to estimate patient’s prognosis [13]. *MYCN* amplification in NB patients is an independent prognostic factor present in about 20 % of all cases. It is strongly related to advanced disease stages, rapid tumor progression and adverse outcome, making this gene an obvious therapeutic target. Being a transcriptional factor, however, it is difficult for pharmacological targeting, and there are currently no clinical trials targeting MYCN protein directly, highlighting the need of alternative approaches.

**Fig. 1:**
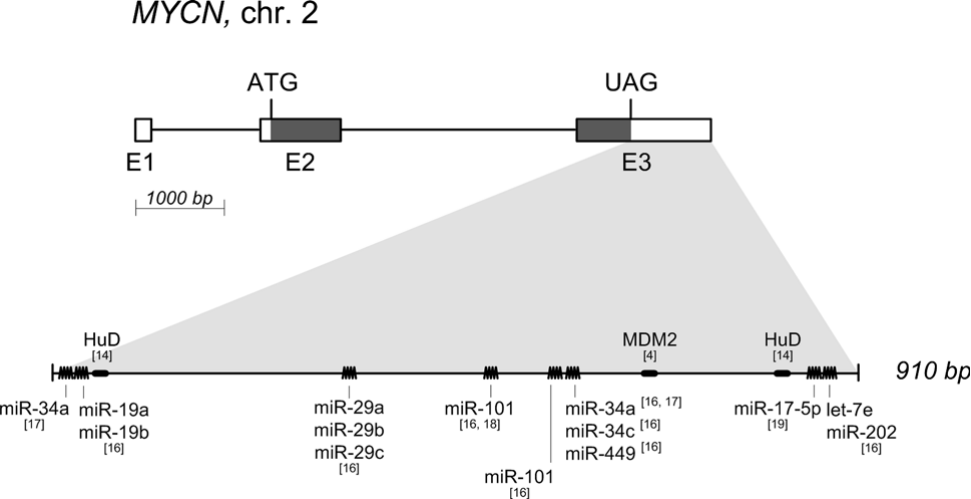
Schematic diagram of the *MYCN* gene. The MYCN transcript (NM_005378.4) is drawn to scale. *Boxes* represent the exons with *shaded regions* corresponding to the CDS,* lines* represent introns. The *lower panel* displays the zoomed-in view of *MYCN* 3′UTR with experimentally validated AREs (*thick lines*) and miRNA target sites (*zigzag lines*). The individual RNA-binding proteins and miRNAs with the corresponding reference sources in *square brackets* are indicated

A detailed computational study of the 3′UTR of the MYCN mRNA demonstrated that it is almost entirely highly conserved in vertebrate phylogenesis. As depicted in Fig. 1, there is an experimental evidence for at least two RBPs (HuD and MDM2) to modulate MYCN mRNA fate through binding to its 3′UTR [4, 14, 15]. It also contains at least eight experimentally validated miRNA binding sites [16–19]. All of this would predict for a highly regulated 3′UTR, therefore making it possible to modulate MYCN protein levels through interferences exerted at its 3′UTR level.

Hence, we focused our efforts on (1) development of a biological model that allows us to investigate the role of *MYCN* 3′UTR in modulating post-transcriptional regulatory processes, and (2) identification of compounds that modify MYCN expression through 3′UTR-dependent control processes.

## Materials and Methods

### Cell Lines

The NB cell lines CHP134, IMR-32, KELLY, LA-N-1, LA-N-2, NB69, SK-N-AS, SK-N-DZ, SK-N-BE(2), and SK-N-SH were obtained from the European Collection of Cell Cultures (ECACC); CHP-126, MHH-NB-11 and SiMa were purchased from Deutsche Sammlung von Mikroorganismen und Zellkulturen GmbH (DSMZ); CHP-212 and SK-N-MC from American Type Culture Collection (ATCC). The cell lines STA-NB-1, -7 and -10 were kindly provided by Dr. Peter F. Ambros from Children’s Cancer Research Institute, Vienna, Austria. All cell lines were cultured in humidified 37 °C, 5 % CO_2_ incubator in a media prepared following the instructions of the suppliers.

### Real-Time Quantitative PCR

*MYCN* copy number status was determined using real-time quantitative PCR (qPCR) approach described elsewhere [20]. Briefly, gDNA was isolated following the protocol of DNeasy Blood & Tissue Kit (Qiagen). qPCR was performed on an Rotor-Gene 6000 real-time system (Corbett Life Science). Each run included a standard curve of four serial tenfold dilution points, normal human DNA (Roche) with a disomic copy number of all genes, gDNA samples from 18 NB cell lines and a no-template control. Amplification mixture (10 μl) for *MYCN*, *BCMA* and *SDC4* contained 5 ng gDNA, 1× Kapa SYBR Fast qPCR Universal Master Mix (2×) (Kapa Biosystems) and 300 nM of corresponding primers (Eurofins MWG Operon) listed in Table S1. The cycling conditions comprised 2 min at 95 °C, 40 cycles at 95 °C for 3 s, 60 °C for 30 s and at 72 °C for 1 s, followed by melting curve analysis. The copy numbers of *MYCN*, *BCMA* and *SDC4* genes in each sample were interpolated from the raw Cq values using the constructed standard curves and then calibrated against a normal human DNA sample. *MYCN* copy number was calculated by dividing the calibrated values by the geometric mean copy number of *BCMA* and *SDC4* reference genes.

Total RNA was purified using RNeasy mini kit (Qiagen) according to the provided protocol. For each sample, cDNA was produced from 1 μg RNA following the protocol from iScript cDNA synthesis kit (BioRad). PCR reactions were performed in a 10 μl reaction volume containing 5 ng template cDNA, 1× Kapa SYBR Fast qPCR Universal Master Mix (2×) and 300 nM of forward and reverse primers (Table S1). All reactions were carried out on a Rotor-Gene 6000 real-time system with the cycling conditions described above. qPCR amplification efficiency was calculated for each gene using a relative standard curve derived from a cDNA mixture of NB samples (a tenfold dilution series with four measuring points). The relative mRNA expression level of each gene was determined in Excel as described elsewhere [21]. The geometric mean of four reference genes (*HPRT1*, *SDHA*, *UBC*, and *GAPDH*) was used for normalization of MYCN expression levels.

### Western Blotting

To analyze MYCN protein level in 18 NB cell lines, total proteins were extracted from pelleted cells in RIPA buffer followed by three freezing/thawing cycles. CHP134 cells treated with CPX for various time periods were harvested and divided in two parts for subsequent total and cytosolic protein isolation using RIPA and Buffer A (10 mM HEPES, 1.5 mM MgCl_2_, 10 mM KCl, 0.5 mM DTT, 0.05 % NP40), respectively. Following quantification with Bradford reagent (Sigma), 10 μg total proteins or 20 μg of cytoplasmic fraction were loaded and electrophoresed using sodium dodecyl sulfate polyacrylamide gel electrophoresis (SDS-PAGE) and transferred to nitrocellulose membranes (BioRad). After blocking, membranes were incubated with anti-MYCN (1:500, OP13, Calbiochem) or anti-GAPDH (1:2000, sc-32233, SantaCruz) as primary antibodies and goat anti-mouse IgG-HRP (SantaCruz) as secondary antibodies. Blots were imaged with ChemiDoc XRS+ imaging system (BioRad) after addition of ECL Select (for MYCN) or Prime (GAPDH) detection reagent (Amersham).

### Reporter Constructs

Reporter constructs containing the Firefly luciferase reporter gene *luc2* under control of a CMV viral promoter and followed by either the whole *MYCN* 3′UTR (pGL4.26-MYCN) or the SV40 poly(A) region (pGL4.26-CTRL) as a control were generated (Fig. S1). pGL4.74 encoding *Renilla* luciferase was purchased from Promega. HuD overexpressing plasmid was kindly provided by Daniele Peroni (CIBIO, Trento, Italy).

### Transient and Stable Transfections

Transient transfection experiments with DNA plasmids were performed in CHP134, KELLY, SK-N-BE(2), and SiMa neuroblastoma cell lines. The cells were seeded in white 96-well plates (Nunc) at 10000, 15000, 20000, and 30000 cells per well, respectively, in 100 μl of complete medium. After overnight incubation at 37 °C and 5 % CO_2_, cells were transfected by addition of the mixture containing TurboFectin 8.0 (0.12–0.3 μl) and plasmid DNA (0.04–0.1 μg) in 3:1 ratio in 10 μl of OPTI-MEM (Gibco). Plasmid DNA represented a 5:1 mixture of either pGL4.26-MYCN or pGL4.26-CTRL plasmids with pGL4.74 encoding *Renilla* luciferase. Luciferase activity was measured by Dual-Glo Luciferase Assay (Promega) according to the protocol of the supplier using a Tecan Infinite M200 plate luminometer. The resulting values of the Firefly luciferase activity were normalized to *Renilla* luciferase activity in the same well. Each different condition was assayed in 3–5 technical replicates and 3 independent biological experiments.

To generate stable clones, CHP134 cells were plated on a 12-well plate and the next day transfected with the mixture containing 100 μl of OPTI-MEM, 2 μg of either pGL4.26-MYCN or -CTRL plasmids and 6 μl of TurboFection 8.0 transfection reagent (OriGene). 24 h post-transfection the cells were trypsinized and transferred to 10 cm dishes. Two days later, the selection of stable clones was started by addition to the growing media of hygromycin B (Life Technologies) at the final concentration of 110 μg/ml. After the stably transfected cells became visible and could be picked, the cells were transferred to a 48-well plate and expanded. All obtained clones were designated as CHP134-MYCN or CHP134-CTRL followed by the number corresponding to a specific clone. Finally, the clones were verified for luciferase activity level using One-Glo luciferase assay (Promega) and for the integrity of CMV promoter and *MYCN* 3′UTR or SV40 late polyA regions using PCR.

miR-34 mimic (Qiagen) and HuD overexpressing plasmid were transfected into CHP134-MYCN#3 cells using modified reverse transfection protocol. 15000 cells were plated in a white 96-well plate (Nunc) in 100 μl growth medium. In about 30 min transfection complexes containing in 20 μl OPTI-MEM either 0.5 μl Lipofectamine RNAiMAX reagent (Life Technologies) and 25 nM miR-34 or 0.45 μl TurboFectin 8.0 and 0.15 μg HuD plasmid were added to the wells with loosely adhered cells. miScript Inhibitor Negative Control (Qiagen) and pEGFP-N1 plasmid (Clontech) were used for mock transfections. Luciferase activity was assayed using One-Glo reagent in 48–72 h.

### Primary Screening and Counter-Screening

The screening represented a cell-based assay of reporter gene type. It was carried out in triplicates in the CHP134-MYCN#3 stable clone with the Spectrum Collection small molecule library (MicroSource Discovery). The library screened consisted of 2000 compounds arranged in 8 rows and 10 columns in 25 96-well plates at the concentration of 10 mM in DMSO. The screening was divided in three runs with the batch size of 27 plates screened in one run. The batch of 27 plates included library plates n.1−n.8+n.25 (first run), n.9−n.16+n.25 (second run) and n.16−n.24+n.25 (third run), each plate screened in triplicate. Thus, the library plate n.25 was assayed within all three runs making possible inter-run comparison.

In each run CHP134-MYCN#3 cells were plated in 27 white 96-well plates (CulturePlate-96, PerkinElmer) at the concentration of 15000 cells per well in 75 μl culture medium using the Tecan EVO 200 robot. Next day the cells were treated for 24 h at the final compound concentration of 2 μM in each well (0.02 % DMSO). Columns 1 and 12 of each plate were used for vehicle-treated controls. Luminescence signal was measured in 30 min after addition of One-Glo luciferase reagent on a Tecan F200 multiplate reader integrated with the robot. Experimental data were pre-processed using normalization of all samples to on-plate vehicle-treated controls. Hits were selected using $\bar{x} \pm 2{\text{SD}}$ as a hit selection threshold, where the mean value $\bar{x}$ and SD are computed over all assay values [22]. The generated hit list was further manually filtered as described in the “Results” section.

The counter-screening was performed for the selected 112 hits, cherry-picked from stock library plates and distributed over two 96-well plates. The assay was carried out in triplicates in CHP134-MYCN#3 and -CTRL#19 cells plated in white (luciferase activity detection) and transparent (viability assay) 96-well plates (PerkinElmer). The cells were grown and treated following the protocol described above. The raw luminescence signals of the samples were normalized to on-plate vehicle-treated controls. In addition to luminescent readout, cell viability was assessed using the WST-1 assay (Roche) according to the protocol of the supplier. Briefly, the WST-1 reagent was added in an amount equal to 10 % of the culture volume and the plates were returned to the incubator. Four hours later the absorbance was measured at 450 nm on the Tecan F200 multiplate reader. The percentage of viable cells was calculated using the following equations: (*T*_i_ − *T*_z_)/(*C* − *T*_z_) × 100 if *T*_i_ ≥ *T*_z_ and (*T*_i_ − *T*_z_)/*T*_z_ × 100 if *T*_i_ < *T*_z_, where *T*_z_ (time zero) is an absorbance measurement at the time of drug addition (performed in separate control plates), control growth (*C*), and test growth (*T*_i_) are absorbance measurements in the presence of a vehicle and a compound of interest, respectively.

## Results

### The *MYCN* 3′UTR Ectopically Affects mRNA Stability in NB Cells

**Fig. 2:**
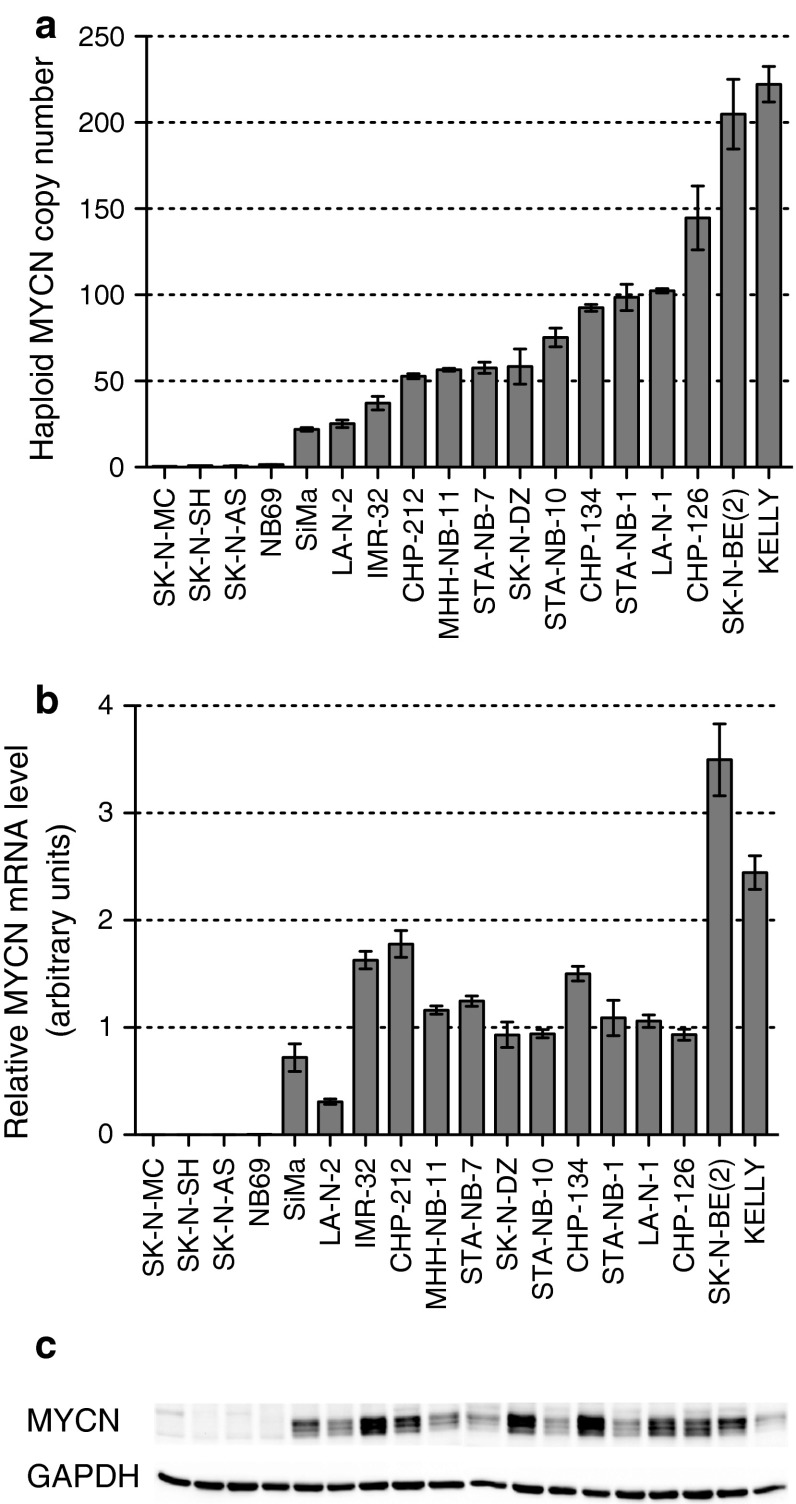
MYCN levels in parental NB cell lines. **a** Haploid *MYCN* DNA copy number obtained by qPCR. The values are reported as normalized to two reference genes (*BCMA* and *SDC4*) and calibrated to a normal human gDNA sample. Mean ± SEM of at least three biological replicates is shown. **b** Relative MYCN mRNA expression levels normalized to geometric mean of four reference genes (HPRT1, SDHA, GAPDH, UBC) as determined by qPCR. Mean ± SEM of at least three biological replicates is reported. **c** The representative western blot showing MYCN and GAPDH protein levels in 18 NB cell lines

*MYCN* amplification found in about 20 % of primary NBs is associated with rapid tumor progression and poor prognosis [13]. In order to set up a cell-based assay addressing the question if deregulated MYCN expression could be modulated through its 3′UTR, the proper cellular model had to be selected. With this in mind, we characterized 18 parental NB cell lines (cell lines directly derived from the original tumor and not by in vitro cell subcloning) in terms of MYCN levels. First, *MYCN* gene amplification was tested using qPCR. As shown in Fig. 2a, 14 out of 18 tested cell lines could be classified as *MYCN*-amplified (MNA), though demonstrating heterogeneous levels of gene amplification. Further, we validated whether *MYCN* amplification indeed resulted in overexpression of its mRNA and finally in comparable production of the protein. In fact, all MNA cell lines demonstrated high MYCN mRNA expression, with a Pearson correlation coefficient of 0.73 (one-tailed *p* value 0.0015). However, when the mRNA levels of MYCN in MNA cells were compared to the corresponding protein levels (Fig. 2b, c), no linear relationship could be found. The lack of correlation between mRNA and protein levels points out to the presence of pronounced post-transcriptional (e.g., mRNA stability, translation efficiency) and/or post-translational (e.g., post-translational modifications leading to variation in the protein degradation rate) mechanisms controlling MYCN expression.

**Fig. 3:**
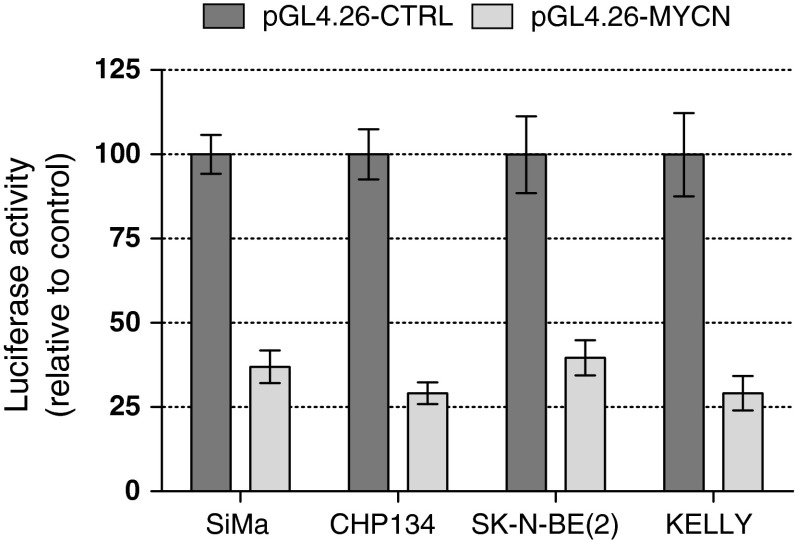
*MYCN* 3′UTR down-regulates expression of a luciferase reporter. The pGL4.26-MYCN and -CTRL plasmids were transiently transfected in SiMa, CHP134, SK-N-BE(2), and KELLY cells as described in “Materials and Methods” section. For each cell line, normalized luciferase activity obtained in transfections with pGL4.26-CTRL plasmid was set to 100 %. The luciferase signal obtained in pGL4.26-MYCN transfections was expressed relative to pGL4.26-CTRL. The mean ± SD of three biological replicates is shown

For initial testing we selected four MNA NB cell lines (SiMa, CHP134, SK-N-BE(2), and KELLY) with different DNA–RNA–protein ratios. To address the role of *MYCN* 3′UTR in the modulation of mRNA fate, at first we produced two reporter constructs. Both constructs contained the Firefly luciferase reporter gene under the control of the CMV promoter followed by either the *MYCN* 3′UTR sequence (pGL4.26-MYCN) or the SV40 poly(A) region (pGL4.26-CTRL) (Fig. S1). Then we tested how and to which extent the presence of the *MYCN* 3′UTR affected the expression of the luciferase reporter. We transiently transfected the two plasmids in the four selected NB cell lines. In all four cases, the introduction of *MYCN* 3′UTR resulted in a decrease of luciferase signal of about 70 % (Fig. 3). The effect was not dependent on the CMV promoter, since similar results were observed using the constructs with the SV40 promoter (data not shown). The effect is also independent from the level of MYCN amplification over a tenfold spectrum (compare Figs. 2a, 3). These data confirmed the existence in a NB cell environment of general (not cell line specific) post-transcriptional control mechanisms exerted through the *MYCN* 3′UTR.

### Development and Validation of the Assay

CHP134 was selected as a final cellular model for the assay for several reasons. CHP134 has been reported to express the antiapoptotic *BCL2* gene at extremely low levels, and to be used as a NB cell death model, i.e., cells would rather undergo apoptosis than neuronal differentiation in response to a treatment [23, 24]. In addition, the intermediate level of MYCN mRNA expression in CHP134 cells compared to SiMa cells from one side and SK-N-BE(2) and KELLY cells from the other side, offered an opportunity of modulation of the mRNA in both directions: down-regulating its expression and up-regulating it. Inhibition of MYCN expression would be desirable because of the “oncogene addiction” concept, i.e., cancer cells become dependent on an activated oncogene, and therefore suppression of the oncogene expression or activity could selectively impair cancer cell survival [25]. Enhancement of MYCN expression might be also detrimental to cancer cells. During the process of tumor development cells raise a barrier to the intrinsic tumor suppression pathways; however, further up-regulation of an activated oncogene capable of inducing programmed cell death might overcome an upper threshold of its tolerance by cancer cells causing death [26].

For the high-throughput format, the luciferase reporter constructs pGL4.26-MYCN and -CTRL were stably integrated in the CHP134 NB cell line. The clones were designated as CHP134-MYCN or CHP134-CTRL, respectively, followed by the number corresponding to a specific clone.

**Fig. 4:**
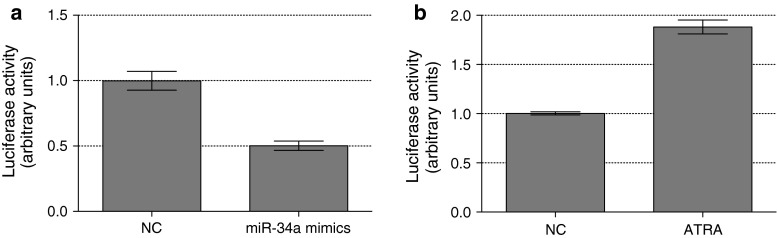
Quality assessment of the assay. CHP134-MYCN#3 clonal cells were plated in a white 96-well plate. **a** Immediately after plating the cells were transfected with 25 nM miR-34a mimics and 25 nM miScript negative control (NC) using modified reverse transfection protocol. **b** The day after seeding the cells were treated with 2 μM all-trans-retinoic acid (ATRA) or vehicle (NC). Luciferase activity was assayed 48 h after seeding using One-Glo reagent (Promega). The graph represents one of two biological replicates, the mean ± SD of eight technical replicates is shown. *Z*-factor calculated from the data is 0.75, suggesting that the assay will perform well in high-throughput format

To determine the feasibility of the assay, we verified if luciferase activity could be up- and down-regulated specifically via the *MYCN* 3′UTR. First, CHP134-MYCN#3 cells were transfected with miR-34a, shown to bind directly to the *MYCN* 3′UTR destabilizing it [17]. The implementation of a modified reverse transfection protocol, i.e., cells were plated immediately before the addition of transfection complexes with miR-34a mimics, allowed us to measure the luciferase activity within the time frame of the screening format. Indeed, luciferase activity assessed 48 h after seeding/transfection was 50 % less in miR-34a transfected cells compared with that of the negative control (Fig. 4a), while no evidence of cytotoxicity was detected under the tested conditions (data not shown). This finding supported the validity of *MYCN* 3′UTR as a functional element able to specifically down-regulate protein production, ectopically in our system. Obtaining a positive control for up-regulation of the reporter activity through *MYCN* 3′UTR turned out to be more complex. Transfection of miR-34a inhibitors into CHP134-MYCN#3 cells did not change luciferase activity, indicating that despite miR-34a is able to mediate its effects when introduced into the cells artificially, it is not *per se* functional in this cell line. As an alternative, the cells were transfected with the plasmid overexpressing HuD, an RNA-binding protein known to stabilize MYCN mRNA through direct binding to its 3′UTR [14]. Significant increase in luminescence was detected after transfection of CHP134-MYCN#3 cells with HuD (Fig. S2). However, this up-regulation could be detected only after 72 h after seeding/transfection and not before, probably due to the time needed for the synthesis of the HuD protein itself. Thus, although we obtained the proof-of-principle increase mediated by HuD, its overexpression could not be used as a screening control due to the longer time between cell plating and assay endpoint. At the time when the described assay was under development, there were no chemical components known to act specifically through *MYCN* 3′UTR. We therefore treated CHP134-MYCN#3 cells with all-trans retinoic acid (ATRA) that up-regulated the reporter activity twice (Fig. 4b) acting most likely through the viral promoter and/or vector sequences and not the 3′UTR, since a similar increase was detected in CHP134-CTRL#19 cells (data not shown). Altogether, the functionality and specificity of the cell-based assay was demonstrated using miR-34 mimics as negative control and HuD as positive control. Including ATRA treatment as positive control allowed us to calculate a *Z*-factor of 0.75 and a signal to background ratio of 3.5 (Fig. 4) indicating robust performance in the high-throughput format.

### Identification of Molecules Modulating MYCN Expression Through Its 3′UTR

**Fig. 5:**
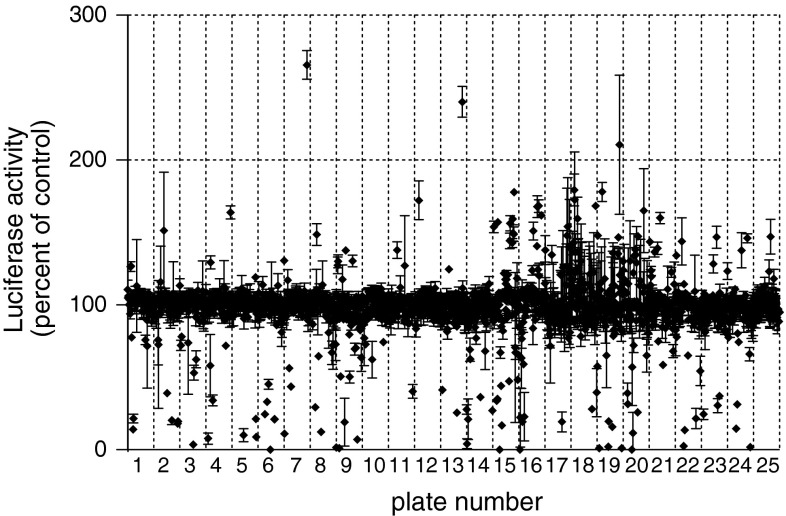
Plate-well scatter plot of normalized values from the primary screening. The screening of 2,000 compounds at 2 μM concentration was carried out in CHP134-MYCN#3 cells.* Each point* corresponds to luciferase activity detected for a single compound after 24 h of treatment and normalized to the mean luciferase signal detected in vehicle-treated controls within the same plate. Mean over three replicates ± SD is shown

A screening aimed at the identification of compounds able to affect deregulated MYCN through its 3′UTR was carried out in the CHP134-MYCN#3 clone using a 2000 compound library (Spectrum collection, Microsource Discovery Systems, Inc.). The library included three main categories of molecules: (i) about 50 % are drugs that have been introduced in the US or are limited in use to Europe and Japan; (ii) about 30 % are natural products; (iii) 20 % are other compounds with known biological activities. The luciferase activity was assessed after 24 h of a 2 μM treatment. Examination of the data from different plates in the screen, depicted in Fig. 5 as fraction of on-plate controls, displayed an overall good screen performance. No evidence of systematic errors manifested through undesirable patterns could be observed. The higher rates of up-regulating hits detected in the library plates n.15−n.21 compared with others apparently reflects a non-random pattern of compounds distribution. In fact, these plates are overpopulated with natural compounds sharing similar molecular structure. Since the screening experiment was carried out in triplicate, the coefficients of variation (CVs) could be calculated for each compound providing an additional measure of performance. The median and mean CVs were 5.0 and 6.5, respectively, indicating a robust assay (Fig. S3a). The day-to-day reproducibility between the runs was also very high, as revealed by comparison across three inter-run replicates of the plate n.25 (Fig. S3b).

**Fig. 6:**
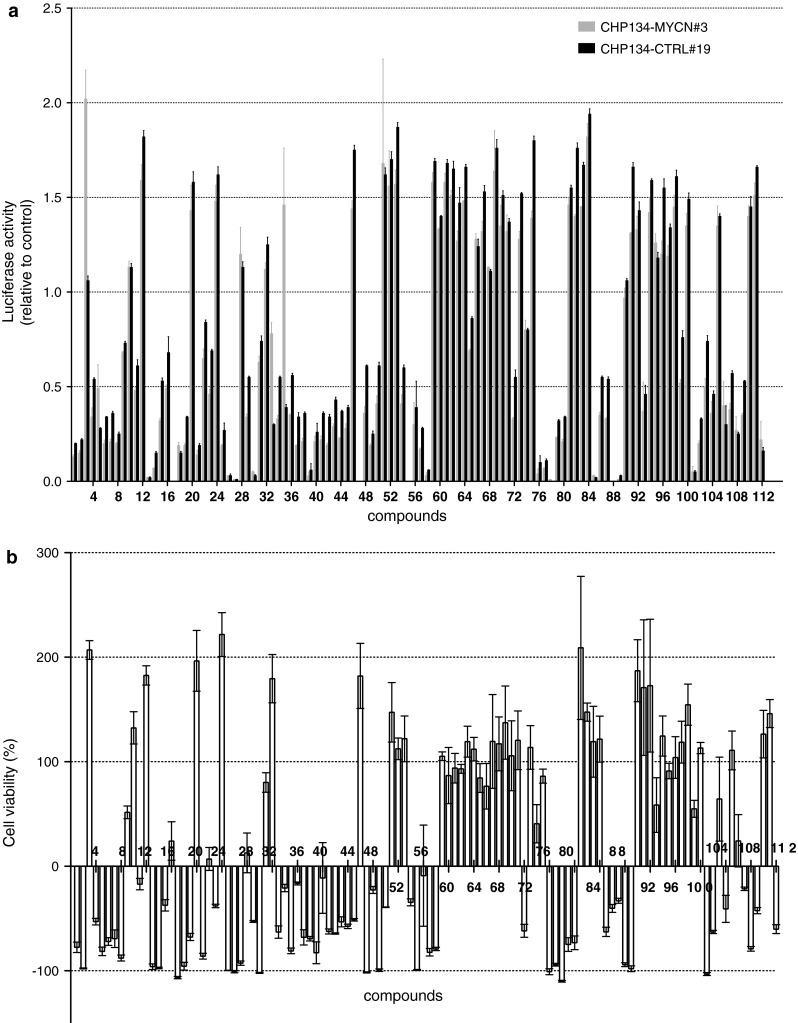
Counter-screening of 112 compounds. **a** Luciferase assay was performed in CHP134-MYCN#3 and -CTRL#19 cells and **b** WST-1 assay in CHP134-MYCN#3 for 112 pre-selected hits, each at 2 μM for 24 h. **a** *Each bar* corresponds to luciferase activity detected for a single compound and normalized to the mean luciferase signal detected in DMSO-treated controls within the same plate. **b** Cell viability was calculated as described in “Materials and Methods” section. Mean over three replicates ± SD is reported

Putting the hit selection threshold above/below two standard deviations from the mean value computed over all assay values, approximately 130 compounds were prioritized. Then, the list was manually filtered favoring FDA-approved drugs over other compounds for potential drug repositioning, and discarding compounds with a general mechanism of cytotoxicity (e.g., pesticides, DNA intercalators, protein synthesis inhibitors). Manual filtering of the primary list prioritized 112 hits (Table S2) that were re-tested and counter-screened for promoter effects (Fig. 6a) and for the phenotypic outcome in terms of cell viability (Fig. 6b).

Overall, the confirmatory screening in CHP134-MYCN#3 cells reproduced very well the results of the primary screening for the 112 selected compounds with a Pearson correlation coefficient of 0.94 (*p* < 0.0001) (Table S2; Fig. S4). The viability WST-1 assay revealed that the majority of the compounds inducing a decreased luciferase activity also displayed cytotoxic effects (Fig. 6). The counter-screening on CHP134-CTRL#19 clonal cells enabled us to discriminate between the hits that caused luciferase up-regulation through the *MYCN* 3′UTR or through the promoter of the reporter plasmid. The counter-screening identified four drugs as truly dependent on *MYCN* 3′UTR, three of which belonged to the anthracyclines class (daunorubicin (N5), doxorubicin (N33), epirubicin (N35)), while the fourth was ciclopirox olamine (CPX) (N3), a synthetic antifungal compound belonging to the hydroxypyridones class.

### Validation of the CPX Hit

**Fig. 7:**
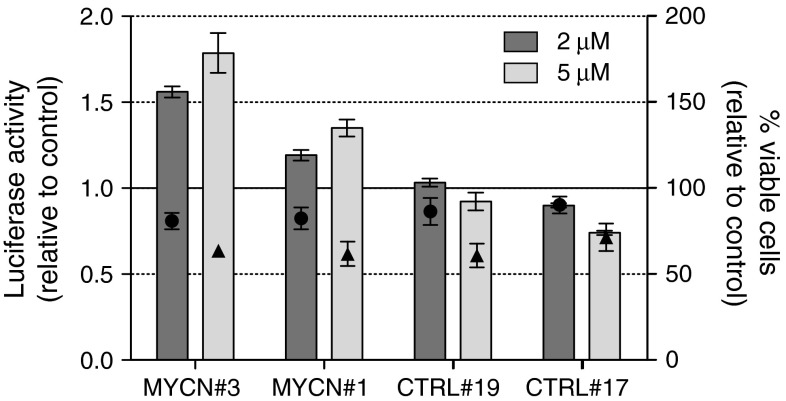
Clone-independency of the CPX effect on *MYCN* 3′UTR. One-Glo luciferase assay was performed in CHP134-MYCN#3, -MYCN#1, -CTRL#19 and -CTRL#17 cells grown in 96-well plates and treated for 24 h with 2 (*dark gray bar*) and 5 μM (*light gray bar*) CPX. Amount of viable cells (Hoechst^+^, PI^−^) upon treatment with 2 μM (*black circles*) and 5 μM (*black triangles*) CPX was counted using high-content imaging system Operetta (PerkinElmer). Data are expressed relative to vehicle-treated controls. Mean ± SEM of three biological replicates is reported

CPX was selected for the final step quality assessment of the assay since it did not compromise the cell viability at the concentration used in the screening and thus did not require any additional experiments for concentration adjustments. To prove dose-dependence of CPX effect for *MYCN* 3′UTR and its clone-independency, the treatment was extended to two concentrations, 2 and 5 μM, in four clones, i.e., CHP134-MYCN#3 and CHP134-CTRL#19 used in the counter-screening and the independent clones CHP134-MYCN#1 and CHP134-CTRL#17. Measurement of luciferase activity with the One-Glo luciferase assay after 24 h proved the clone-independency of the results obtained before (Fig. 7). Moreover, the effect of increased luciferase expression became even more evident upon treatment with 5 μM CPX.

Though the values of the WST-1 assay demonstrated about twofold increase in cell viability (Fig. 6b), no obvious difference in cell number could be observed under microscope as compared to vehicle-treated controls. This discrepancy was addressed by counting viable cells in all clones, either vehicle-treated or treated with 2 and 5 μM CPX. As shown in Fig. 7, 2 μM CPX had very little effect on CHP134 cell viability in 24 h, whereas treatment of cells with 5 μM CPX decreased number of viable cells to about one-half of the control. Taken together, it is possible to conclude that CPX elicited a clone-independent, *MYCN* 3′UTR specific effect on increased luciferase expression in CHP134 NB cells.

Finally, we investigated whether the effect of CPX on the *MYCN* 3′UTR detected by reporter cell-based assay could be recapitulated under physiological conditions, i.e., if CPX treatment was able to modulate MYCN protein levels. Given the high abundance of MYCN protein in CHP134 cells (Fig. 2), this substantial background might render visualization of slight differences in total protein levels very difficult. Therefore, taken into account that MYCN protein has mostly nuclear localization, the differences in de novo synthesized protein could be appreciated better by separation of the cytoplasmic fraction from the rest.

**Fig. 8:**
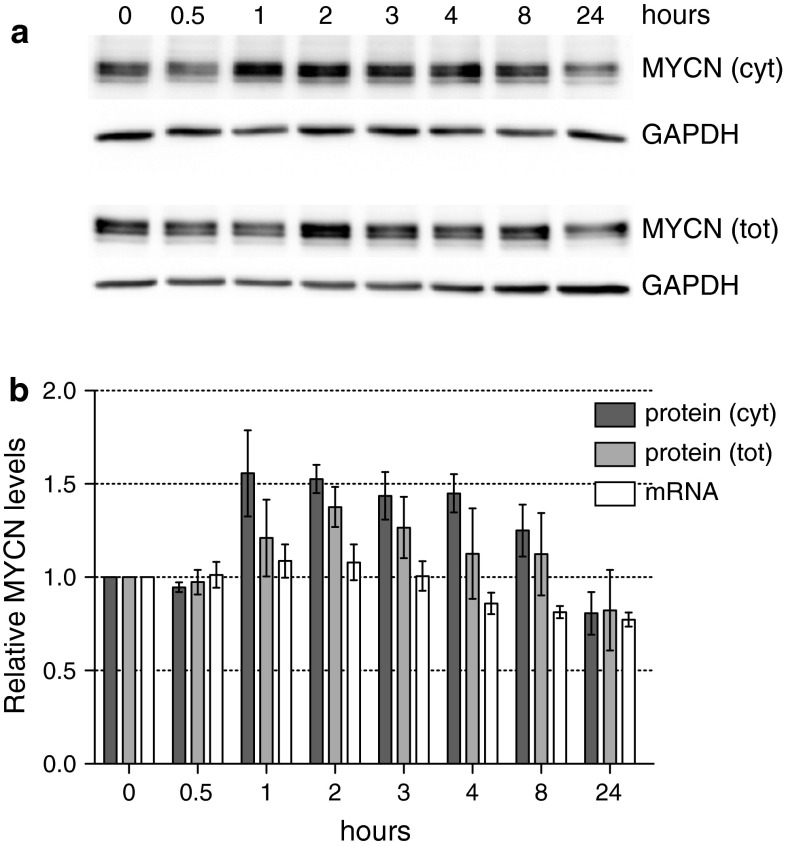
The effects of CPX treatment on MYCN levels. CHP134 cells treated with 2 μM CPX were harvested at specified time points. For each condition RNA and protein lysates were prepared. 20 μg of cell cytoplasmic or 10 μg of total protein lysates was loaded and electrophoresed using SDS-PAGE and transferred to nitrocellulose membrane. Proteins were detected using anti-MYCN and anti-GAPDH. **a** The representative western blots of three biological replicates are displayed. **b** The *bar graph* shows quantification of MYCN protein after normalization using the levels of GAPDH protein; cytoplasmic MYCN levels are represented by *dark gray bars*, total MYCN by *light gray bars*.* White bars* correspond to relative MYCN mRNA expression levels normalized to geometric mean of HPRT1 and SDHA reference genes as determined by qPCR. Mean ± SEM of three biological replicates is reported

Following the outline of the screening, the parental CHP134 cells were treated with 2 μM CPX for 24 h followed by western blot analysis. Surprisingly, the treatment resulted in down-regulation of MYCN protein expression and not in its up-regulation as predicted by the screening results. Taking into account the half-life time of MYCN protein of about 30 min, earlier time points were verified. In fact, an increase in MYCN protein could be detected as short as 1 h after initiation of the treatment with a peak around 2 h and a final decrease by 24 h (Fig. 8). The apparent inconsistency in timing of CPX effects revealed by western blot analysis and by the reporter assay was clarified after evaluation of the stability of the luciferase protein. Firefly luciferase in CHP134 cells turned out to have a half-life time of more than 9 h (data not shown), allowing for the detection of the early events even after 24 h.

To rule out the possibility that up-regulation of the MYCN protein is a consequence of transcriptional changes, we performed qPCR analysis. In agreement with the data of the screening indicating a post-transcriptional mode of CPX effects on MYCN, qPCR analysis revealed the lack of a concomitant increase in MYCN mRNA levels within the first hours of CPX treatment. This result indicated that CPX treatment did not affect the stability of the MYCN transcript either, thus excluding this mechanism of post-transcriptional regulation as the cause of the MYCN protein increase.

To sum up, the developed cell-based reporter gene assay allowed us to identify molecules modulating protein levels via post-transcriptional mechanisms dependent on a 3′UTR. Moreover, we successfully extrapolated CPX-mediated *MYCN* 3′UTR-dependent up-regulation of luciferase on endogenous MYCN protein expression. The increased MYCN expression upon CPX treatment could most likely result from enhanced translational efficiency of its mRNA, due to changed polysomal loading and/or ribosomal occupancy of the MYCN transcript. Further studies are required to unveil the exact molecular mechanism underlying the reported effect.

## Discussion

MYCN is together with MYC and MYCL a member of the MYC family of transcription factors that have roles in many if not all aspects of cell fate including cell proliferation and growth, survival and differentiation [27]. It is therefore not surprising that deregulation of MYC proteins contributes to the development of a wide variety of human cancers. In particular, the amplification of *MYCN* gene is the most common focal genetic lesion in sporadic NB. It occurs in about 22 % of NB tumors and is associated with advanced stages of disease, rapid tumor progression and poor prognosis [13]. *MYCN* amplification and consequent overexpression has been also found in other cancers of neural origin, including glioblastoma, medulloblastoma, retinoblastoma, small cell lung carcinoma, peripheral neuroectodermal tumors [28].

Despite deregulation of MYCN is a marker of high-risk NB, there are currently no clinical trials aiming the MYCN protein directly [11]. Transcription factors including MYCN itself have traditionally been considered as very difficult for pharmacological targeting, since their function is largely mediated through protein–protein interactions rather than enzymatic activities, and it is difficult to design specific small molecules to interrupt these large surface interactions. Alternative pharmacological approaches to deal with the deregulated MYCN expression are focused on its mRNA, with the aim of either suppressing its production or favoring its degradation. The best-known example of a compound able to decrease MYCN mRNA production is retinoic acid (RA) [29]. Transcriptionally mediated down-regulation of *MYCN* is a pivotal event in RA-induced differentiation of NB cells. Indeed, the use of RA is currently part of a protocol used for the treatment of high-risk NB [30]. As to the second strategy, selective knocking down of a gene’s expression through sequence-specific binding of RNA molecules is of particular interest in drug discovery and development. Knockdown of MYCN expression with antisense oligonucleotides in MNA NB has been reported to decrease tumorigenesis in mouse model underlying the potential of this strategy for NB therapy [31].

In this report we focus on yet another approach to address deregulated MYCN expression, namely to target its mRNA fate via modulation of post-transcriptional control mechanisms. The fact that examination of 14 MNA NB cell lines for MYCN status revealed a lack of correlation between mRNA and protein levels supports the functional importance of these control mechanisms for MYCN deregulated expression (Fig. 2). Post-transcriptional regulation of MYCN expression is largely exerted through the *MYCN* 3′UTR as can be predicted by the high level of its conservation in vertebrate phylogenesis. In particular, the *MYCN* 3′UTR influences the gene expression via modulation of its mRNA decay. This effect is largely mediated by *cis*-acting destabilizing elements residing within the 3′UTR sequence [14]. In addition, several miRNAs affect MYCN protein levels via interaction with its 3′UTR, resulting in translational repression [16–19, 32]. Also in the NB cells tested, the *MYCN* 3′UTR was shown to affect mRNA fate, since its introduction to the reporter plasmid reduced luciferase activity to 30–40 % of the control value (Fig. 3). Moreover, in CHP134 cells both mRNAs, the endogenous *MYCN* and the luciferase reporter followed by *MYCN* 3′UTR, showed a similar half-life time of about 30 min (data not shown) further supporting the functionality of the 3′UTR sequence.

To identify compounds able to modulate MYCN mRNA fate via mechanisms dependent on its 3′UTR, the stable cell line CHP134-MYCN was generated and characterized as a sensitive and reliable luciferase reporter assay. Using this approach changes in luciferase activity serve as indirect measure of affected mRNA stability and/or translation efficiency. *MYCN* 3′UTR conferred responsiveness to the luciferase reporter following transient transfection of miR-34a mimic and HuD overexpressing plasmid, thus validating the model. Application of this cellular model for testing 2000 compounds produced over 100 potential hits, both inhibitors and stimulators of luciferase activity. The counter-screening of 112 selected compounds allowed to subdivide all of them in two groups according to viability data. Compounds of the first group caused an efficient cytotoxicity in CHP134 cells, while the other group had no significant impact on viability under the settings used in the screening. Comparison between luciferase activity and the corresponding cell viability data displayed very strong correlation between the two (Pearson coefficient of 0.907 with *p* value < 0.0001). In other words, significant decrease of luciferase activity was largely a consequence of cytotoxic effects. In this context, specific effects of *MYCN* 3′UTR could be overlooked for the compounds with pronounced cytotoxicity resulting in false negative hits. That could account for a failure to detect down-regulating hits truly dependent on the *MYCN* 3′UTR. Further titration would be necessary for the compounds that at 2 μM concentration caused significant reduction in viability. This would allow us to select an appropriately lower concentration in order to discriminate if a compound of interest exert a specific effect on the *MYCN* 3′UTR.

Recently Frenzel et al. [33] screened 80 conventional cytotoxic drugs in order to identify the compounds whose effect on cell viability could be potentiated by MYCN overexpression. From 19 drugs shown to be more effective in the MYCN overexpressing cells, 13 were present in the Spectrum Collection library and 12 were selected in our primary screening for the counter-screening. The 13th drug cycloheximide, in fact, also passed the threshold for hit selection, but was not followed in the counter-screening based on our previous knowledge about its effective concentration in CHP134 cell model. This striking overlap highlights a validity of the chosen cellular model.

Another valuable information was retrieved by clustering the hits based on chemical and functional similarity. The largest inhibitor group was composed by chemotherapeutic drugs including three of six drugs commonly used for treatment of NB (vincristine, etoposide and doxorubicin). Of note, verification why the remaining three drugs (topotecan, cyclophosphamide and cisplatin) were not counter-screened revealed that one of them, topotecan, is not present in the Spectrum collection library. Cyclophosphamide is a prodrug, i.e., it should be converted to its active form through the normal metabolic processes of the body. Finally, cisplatin was the only of the six compounds to which CHP134 cells were quite resistant, demonstrating IC50 values in the micromolar range (data not shown).

The whole class of cardiac glycosides included in the Spectrum Collection library (13 compounds) was represented in the hit list. Cardiac glycosides are a family of naturally derived compounds that bind to and inhibit the sodium pump. The increased susceptibility of cancer cells to these compounds supports their potential use as antitumor agents, and the first generation of glycoside-based anticancer drugs have now entered clinical trials for treating cancer [34]. There is some preclinical evidence for the anticancer properties of cardiac glycosides in NB. Kulikov et al. [35] demonstrated in vitro that ouabain triggers a variety of signaling pathways ultimately leading to NB cell death. Svensson et al. [36] showed that digoxin causes a specific NB growth inhibition in mice grafted with two NB cell lines, SH-SY5Y and Neuro-2a.

Finally, most of the hits endowed with up-regulating luciferase activity belonged to a big group of natural compounds called flavonoids. However, detection of increased luciferase activity upon treatment with flavonoids in both CHP134-MYCN and -CTRL cells indicated that this increase was not due to the presence of the 3′UTR sequence. The most prominent up-regulation of luciferase activity was detected upon treatment with tretinoin (ATRA) and isotretinoin (13-cis RA). RA exerts its activity by binding distinct members of the retinoid superfamily of nuclear receptors, which have been reported to affect luciferase expression from viral promoters [37]. This effect was dependent on both viral promoter and luciferase reporter itself. Also other flavonoids, given their high degree of structural similarity with RA, are very likely to mediate their effects through the CMV viral promoter and/or some sequences within the *luc2* gene. Up-regulation of the luciferase signal by flavonoids turned out to be not specific, resulting in a high rate of false positive hits. Treating the cells with a transcription inhibitor before drug addition would help to discriminate if the changes in luciferase signal are due only to the effects mediated through the 3′UTR or are the consequence of transcriptional events. While this option is to be considered for shorter treatments (hours), caution should be taken for screenings with prolonged incubation times (24 h or more) since transcriptional blocking itself would have deleterious effects on cells.

Despite the fact that the rationale of the screening was to identify compounds able to down-regulate MYCN expression so reducing the oncogenic potential of MYCN, 4 compounds selected in the screening were able to up-regulate MYCN, but inducing efficient cytotoxicity as well (Fig. 6). A possible reason of this paradoxical outcome could rely on two opposite functions exhibited by the MYC family proteins. MYC proteins promote cell growth and proliferation, however their activation provokes intrinsic safeguard mechanisms including apoptosis, cellular senescence and DNA damage response [27]. During the process of tumor development, proliferation needs to be uncoupled from apoptosis [26]. This barrier is overcome under the form of a variety of mutations, which inactivate coordinated networks responsible for cell death thus shifting the equilibrium toward anti-apoptotic determinants. The compensating mutations accumulated during tumorigenesis will determine an upper threshold of tolerance for the oncogene. Therefore, additional over-activation of the already activated oncogene such as MYCN could determine a massive overcoming of the barriers resulting in extinction of the cells bearing the activated oncogene.

Among the 4 molecules selected as truly dependent on *MYCN* 3′UTR, 3 are well-known chemotherapeutic agents belonging to the anthracycline class, which is used in the first line treatment of NBs (doxorubicin, daunorubicin and epirubicin). The fourth molecule is a topically used antifungine compound, CPX. CPX as well as a big proportion of other compounds in the Spectrum Collection library are FDA-approved drugs with already well-established pharmacodynamics and pharmacokinetics. Finding new applications for these compounds would provide a shorter pathway to clinical trials for treatment of other diseases. This drug repositioning strategy is increasingly used since it not only decreases times to the clinic but also significantly reduces the costs and improves success rates. Though originally developed as antimicotic agent, CPX has recently been demonstrated to be an effective anti-tumor agent for the treatment of leukemia and myeloma cells in vitro [38]. An ongoing study is evaluating anti-cancer properties of CPX in patients with relapsed or refractory hematologic malignancy (http://www.clinicaltrials.gov). To our knowledge, CPX has not been investigated in relation to NB yet. It would be of interest to investigate the potential of CPX as NB therapeutic agent.

In conclusion, we described a biological model that enables the identification of compounds modulating the level of a protein of interest by specifically targeting post-transcriptional control mechanisms. Post-transcriptional regulation represents a mechanism of biomedical interest with a potential for pharmacological intervention. This approach offers several advantages. First, it creates novel biological targets for drug design potentially resulting in ability to treat diseases with no cure at the moment. Second, it allows to screen for mechanisms not only inhibiting the protein production but also increasing the levels of the targeted proteins. Third, perturbing post-transcriptional gene expression controls offers an alternative approach for difficult-to-target proteins.

## Electronic supplementary material

Below is the link to the electronic supplementary material.

Supplementary material 1 (DOCX 17 kb)

Fig. S1 Supplementary material 2 (EPS 70 kb)

Fig. S2 Supplementary material 3 (EPS 97 kb)

Fig. S3 Supplementary material 4 (EPS 479 kb)

Fig. S4 Supplementary material 5 (EPS 113 kb)

Table S1 Supplementary material 6 (PDF 31 kb)

Table S2 Supplementary material 7 (XLS 49 kb)
