# Supplementary material for: A Cell-Based High-Throughput Screen Addressing 3′UTR-Dependent Regulation of the MYCN Gene
Source: Mol Biotechnol. 2014 Feb 11;56(7):631–43. doi: 10.1007/s12033-014-9739-z (PMC4067544; doi:10.1007/s12033-014-9739-z)
Supplement: Supplementary file 1 — Supplementary material 1 (DOCX 17 kb) [file 12033_2014_9739_MOESM1_ESM.docx]

**SUPPLEMENTARY MATERIAL**

**Figure S1. Schematic representation of Firefly luciferase reporter constructs.**

(A) pGL4.26-MYCN. (B) pGL4.26-CTRL. CMV, cytomegalovirus; SV40, simian virus 40; UTR, untranslated region.

**Figure S2. Effect of HuD overexpression on luciferase reporter carrying *MYCN* 3'UTR.**

CHP134-MYCN#3 cells were seeded in a white 96-well plate. Transfection complexes containing pEGFP-N1 (indicated as NC) or HuD overexpressing plasmid were added to the cells immediately after plating. Luciferase activity was assayed 72 hours after seeding using One-Glo reagent (Promega). The graph represents the mean ± SD. Asterisks indicate statistical significance in two-tailed t-test, *** P<0.001.

**Figure S3. Evaluation of the screen performance.**

(A) Density plot displays the percentage of relative frequency distribution of coefficients of variation (CV). CV was computed for each condition from raw luciferase data and analyzed for frequency distribution with the bin width of 1%. The CV values larger than 20 % were included into analysis but omitted from the plot. (B) Inter-run comparison of the data obtained for each of 80 compounds arrayed in the library plate n.25. Every point represents luciferase activity (as percent of control) measured in a well treated with a single compound in run 1 (black asterisk), run 2 (grey square) and run 3 (white diamond).

**Figure S4. Reproducibility of the primary screening for the selected 112 hits.**

The scatterplot summarizes the results of luciferase activity obtained for 112 selected compounds in primary and confirmatory screenings. The reported values represent luciferase activity measured upon treatment with a specified compound and normalized to the mean of vehicle-treated controls in the same plate.

**Table S1. qPCR primers.**

**Table S2. The list of 112 compounds selected for counter-screening.**
