# Supplementary material for: A Cell-Based High-Throughput Screen Addressing 3′UTR-Dependent Regulation of the MYCN Gene
Source: Mol Biotechnol. 2014 Feb 11;56(7):631–43. doi: 10.1007/s12033-014-9739-z (PMC4067544; doi:10.1007/s12033-014-9739-z)
Supplement: Supplementary file 6 — Supplementary material 6 (PDF 31 kb) [file 12033_2014_9739_MOESM6_ESM.pdf]

Gene	Application	Primer	Sequence
MYCN	GCN	forward	5'-CGCAAAAGCCACCTCTCATTA-3'
		reverse	5'-TCCAGCAGATGCCACATAAGG-3'
BCMA	GCN	forward	5'-CGACTCTGACCATTGCTTTCC-3'
		reverse	5'-AAGCAGCTGGCAGGCTCTT-3'
SDC4	GCN	forward	5'-CAGGGTCTGGGAGCCAAGT-3'
		reverse	5'-GCACAGTGCTGGACATTGACA-3'
MYCN	GXP	forward	5'-CCGGGCATGATCTGCAA-3'
		reverse	5'-CCGCCGAAGTAGAAGTCATCTT-3'
SDHA	GXP	forward	5'-TGGGAACAAGAGGGCATCTG-3'
		reverse	5'-CCACCACTGCATCAAATTCATG-3'
HPRT1	GXP	forward	5'-TGACACTGGCAAAACAATGCA-3'
		reverse	5'-GGTCCTTTTCACCAGCAAGCT-3'
UBC	GXP	forward	5'-ATTTGGGTCGCGGTTCTTG-3'
		reverse	5'-TGCCTTGACATTCTCGATGGT-3'
GAPDH	GXP	forward	5'-TGCACCACCAACTGCTTAGC-3'
		reverse	5'-GGCATGGACTGTGGTCATGAG-3'

GCN=gene copy number quantification
GXP=gene expression quantification
